# CCAAT/Enhancer binding protein β induces motility and invasion of glioblastoma cells through transcriptional regulation of the calcium binding protein S100A4

**DOI:** 10.18632/oncotarget.2976

**Published:** 2015-01-24

**Authors:** Diana Aguilar-Morante, Jose A. Morales-Garcia, Angel Santos, Ana Perez-Castillo

**Affiliations:** ^1^ Instituto de Investigaciones Biomédicas, (CSIC-UAM), Departamento Modelos Experimentales de Enfermedades Humanas, Arturo Duperier, 4, 28029-Madrid, Spain; ^2^ Centro de Investigación Biomédica en Red sobre Enfermedades Neurodegenerativas (CIBERNED), Spain; ^3^ Departamento de Bioquímica y Biología Molecular, Facultad de Medicina, Universidad Complutense de Madrid, 28040-Madrid, Spain; ^4^ Instituto de Biomedicina de Sevilla, IBiS, (Hospital Universitario Virgen del Rocío/CSIC/Universidad de Sevilla), Departamento de Fisiología Médica y Biofísica, 41013-Sevilla, Spain

**Keywords:** C/EBPβ, S100A4, invasiveness, transcriptional activity, cancer stem cells

## Abstract

We have previously shown that decreased expression of CCAAT/Enhancer binding protein β (C/EBPβ) inhibits the growth of glioblastoma cells and diminishes their transformation capacity and migration. In agreement with this, we showed that C/EBPβ depletion decreases the mRNA levels of different genes involved in metastasis and invasion. Among these, we found S100 calcium binding protein A4 (S100A4) to be almost undetectable in glioblastoma cells deficient in C/EBPβ. Here, we have evaluated the possible role of S100A4 in the observed effects of C/EBPβ in glioblastoma cells and the mechanism through which S100A4 levels are controlled by C/EBPβ. Our results show that C/EBPβ suppression significantly reduced the levels of S100A4 in murine GL261 and human T98G glioblastoma cells. By employing an S100A4-promoter reporter, we observed a significant induction in the transcriptional activation of the S100A4 gene by C/EBPβ. Furthermore, overexpression of S100A4 in C/EBPβ-depleted glioblastoma cells reverses the enhanced migration and motility induced by this transcription factor. Our data also point to a role of S100A4 in glioblastoma cell invasion and suggest that the C/EBPβ gene controls the invasive potential of GL261 and T98G cells through direct regulation of S100A4. Finally, this study indicates a role of C/EBPβ on the maintenance of the stem cell population present in GL261 glioblastoma cells.

## INTRODUCTION

Glioblastomas (GBM) constitute approximately 50% of gliomas as well as 20% of intracranial tumors. These tumors are characterized by uncontrolled cell proliferation, necrosis focus, high angiogenic activity, apoptosis resistance and diffuse invasion of neoplastic cells into the surrounding tissue, making surgical interventions rather ineffective [1]. GBMs represent the most malignant type of astrocytomas and, despite advances in treatment, they remain refractory to conventional therapies and the medium survival of patients is only one year after diagnosis [2]. This poor prognosis is probably due, at least in part, to the infiltrative nature of these tumors and the presence of cellular populations with ability to escape therapies and drive tumor recurrence and progression. In some cases, these resistant cells exhibit stem properties (Glioblastoma Stem cells (GSC) [3].

CCAAT/Enhancer Binding Protein β (C/EBPβ), is a member of a family of transcription factors consisting of six structurally related basic leucine-zipper DNA-binding protein [4]. C/EBPβ has important roles in numerous tissues regulating cellular proliferation and differentiation, metabolism, adipogenesis, inflammation, transformation and tumorigenesis [5, 6]. This protein is expressed in the central nervous system where it plays different roles [7, 8]. Our group has shown that C/EBPβ is an important factor for neuronal differentiation [9] and regulates the expression of several genes involved in inflammatory processes and brain injury [10]. Besides, mice lacking C/EBPβ show a reduced inflammatory response after an excitotoxic insult and are less susceptible to neuronal cell loss [11].

The effects of C/EBPβ in tumor development is controversial; some evidence suggests that C/EBPβ acts as potent promoter of tumorigenesis while others indicate that it can have antiproliferative effects. For instance, ectopic expression of C/EBPβ in primary fibroblast causes cell cycle arrest and is required for Ras^V12^-induced senescence [12]. By contrast, it has been observed that C/EBPβ is abundant in colorectal tumors [13] and ovarian cancer [14] and its expression is associated with tumor progression. Recently, some data show that C/EBPβ is associated with tumor progression in prostate cancer cells and regulates the expression of metastatic genes in these tumors [15, 16]. Regarding brain tumors, Homma et al. (2006) has shown that expression of C/EBPβ is increased in high grade gliomas compared with less aggressive gliomas, and patients where the expression of C/EBPβ is lower have a longer survival [17]. In this regard, we have demonstrated that C/EBPβ plays an important role in the regulation of proliferation, migration and invasiveness of glioblastoma cells [18]. Furthermore, our results showed that a decrease in C/EBPβ expression is associated with reduced mRNA levels of different genes involved in invasiveness and metastasis, including S100A4 [18].

S100A4, also known as mts1, is a member of the S100 family of Ca2^+^-binding proteins and is thought to be directly involved in tumor invasion and metastasis via interactions with specific protein targets [19] in a variety of tumors, such as breast cancer [20, 21], gastric cancer [22], pancreatic cancer [23], colorectal cancer [24], non-small cell lung cancer [25], and prostate cancer [26]. Recent data show that expression of S100A4 in tumors of the central nervous system is related to the degree of malignancy of the tumor, with a higher expression of S100A4 in high-grade gliomas compared to low-grade gliomas [27–29].

All these data together prompted us to analyze whether C/EBPβ is a direct regulator of S100A4 expression and if this protein could mediate the observed effects of C/EBPβ on migration and invasiveness of glioblastoma cells. Also, we have studied the effects of C/EBPβ depletion on the neurosphere formation of mouse glioblastoma GL261cells.

Collectively, these studies show that C/EBPβ increases S100A4 levels by directly activating S100A4 promoter expression in glioblastoma cells and provide evidence that S100A4 may contribute to the C/EBPβ-induced invasiveness. Also, we demonstrate that S100A4 promotes migration and invasiveness of glioblastoma cells. Finally, our results indicate that C/EBPβ depletion resulted in an inhibition of proliferation and self-renewal of these cells, which is accompanied by a decreased in the S100A4 protein levels.

## RESULTS

### Regulation of S100A4 gene expression by C/EBPβ transcription factor

**Figure 1:**
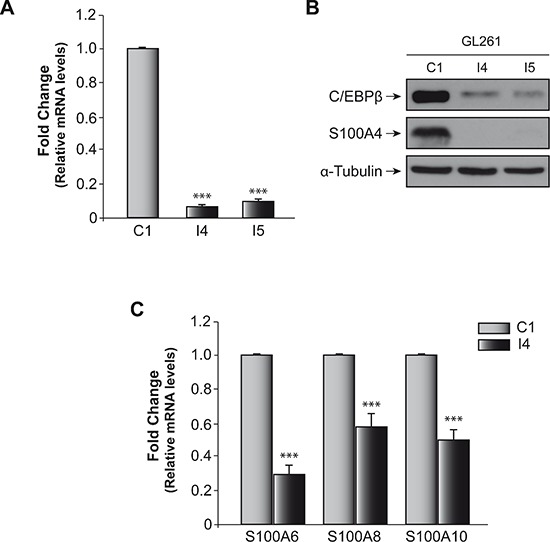
Effect of C/EBPβ on S100A4 expression in glioblastoma murine GL261 cell line **(A)** Quantification of S100A4 mRNA levels in GL261 control cell line (C1) and C/EBPβ-depleted (I4, I5) cells by quantitative real time-PCR. As indicated in Methods, we used Taqman probes specifics to S100A4 and β-Actin mouse. The graphic shown the means of values 2^−ΔΔCt^ of S100A4/β-actin ± SD. ***p < 0.001. **(B)** Representative Western blot showing expression of C/EBPβ and S100A4 in C1, I4 and I5 cell lines. **(C)** Quantification of S100A6, S100A8 and S100A10 mRNA levels in GL261 control cell line (C1) and C/EBPβ-depleted (I4) cells by quantitative real time-PCR. As indicated in Methods, we used Fast SYBR Green and primers specifics to S100A6, S100A8, S100A10 and mouse β-Actin. The graphic shown the means of values 2^−ΔΔCt^ ± SD. ***p < 0.001.

In a previous work [18] we screened two arrays of cell cycle and cancer-related genes in order to analyze the mechanism involved in the tumorigenic effect of C/EBPβ in glioblastoma cells. We identified a number of genes involved in different processes, such as cell cycle regulation, DNA damage response, adhesion, invasion, and metastasis to be differentially expressed in those cells deficient in C/EBPβ. Among these genes, S100A4 showed extremely low levels of expression in the I4 cells. Here we have deeply analyzed the regulation of this gene by C/EBPβ. Figure 1A shows that the levels of S100A4 mRNA, as measured by RT-PCR were significantly decreased (16- and 15-fold) in those cells deficient in C/EBPβ (pools I4 and I5, respectively) compared to controls (pool C1). In addition to this reduction in the mRNA levels of S100A4, S100A4 protein levels were almost undetectable in these cells (Figure 1B). These data are consistent with the PCR arrays data suggesting a possible link between C/EBPβ and S100A4 during the progression of the tumorigenic capacity of glioblastoma cells. We next tested whether other members of the S100 family, also involved in tumorigenic processes, were also regulated by C/EBPβ. As can be seen in Figure 1C, the expression of S100A6, A100A8, and S100A10 was also down-regulated in those glioblastoma cells deficient in C/EBPβ (pool I4).

### C/EBPβ is a direct transcriptional regulator of S100A4

**Figure 2:**
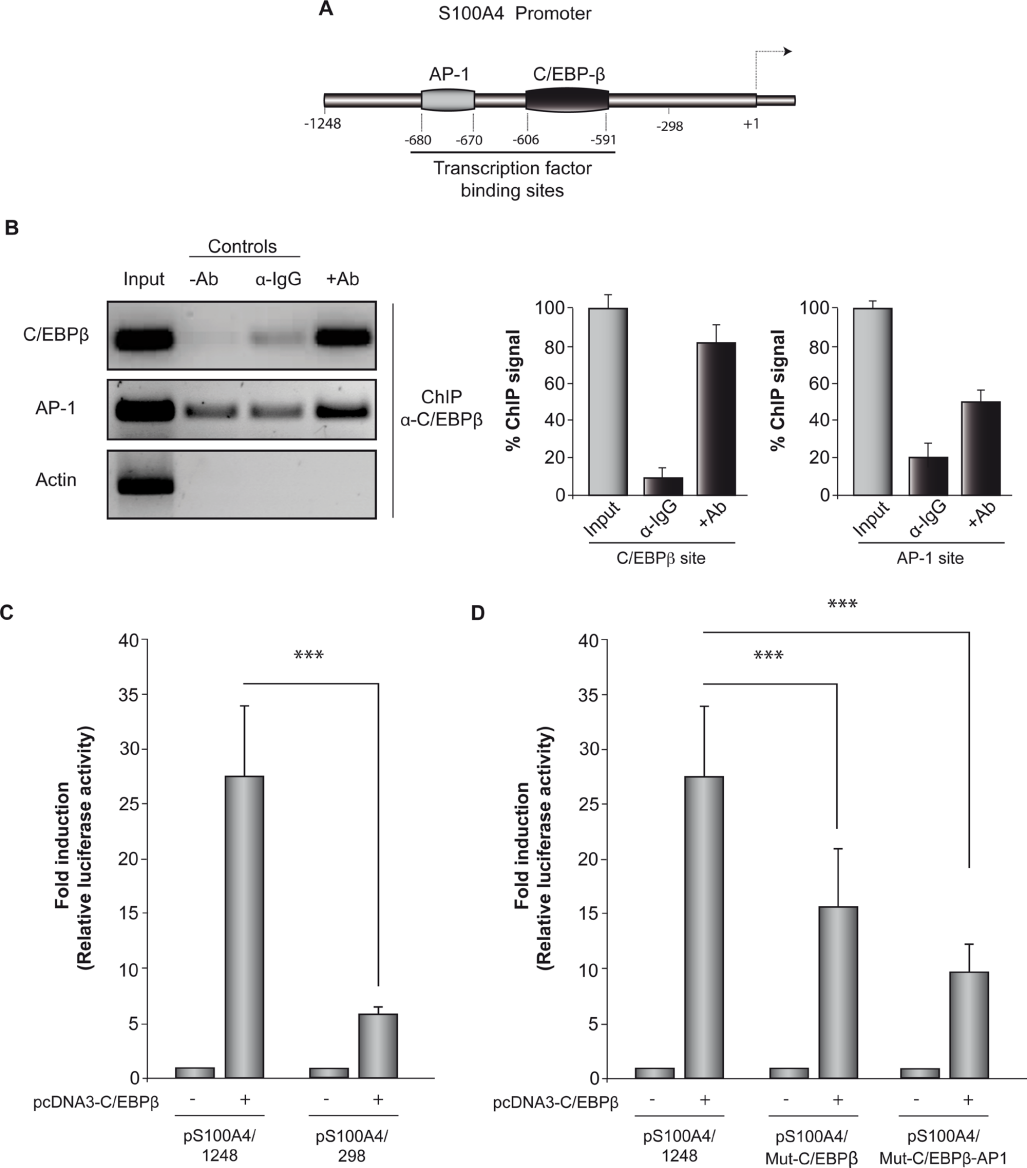
C/EBPβ actives S100A4 promoter **(A)** Schematic diagram of S100A4 promoter showing the localization of C/EBPβ and AP1 binding sites. **(B)** Representative image and quantification of ChIP analysis of C/EBPβ binding to the endogenous S100A4 promoter in GL261 cells. DNA before immunoprecipitation was used as positive control (Input) and a region in the actin gene was used as a negative control. Data are expressed relative to the input values and represent the mean ± SD determined in three independent experiments. **(C)** and **(D)** Transient transfection experiments. The entire promoter fragment (pS100A4/1248), a 5′-deletion construct (pS100A4/298), and two constructs containing the mutated C/EBPβ (pS100A4/Mut-C/EBPβ) and the mutated C/EBPβ and AP1 binding sites (pS100A4//Mut-C/EBPβ-AP1) were created as indicated in Methods. Data are expressed relative to the basal values and represent the mean ± SD luciferase activity determined in triplicate in at least three independent experiments. ***p < 0.001.

To further analyze the role of C/EBPβ in regulating S100A4 gene expression, we studied whether C/EBPβ regulates S100A4 promoter activity. First, we performed an *in silico* analysis to search for putative C/EBPβ binding sites in the S100A4 promoter using TFSEARCH and MatInspector programs. We identified a consensus binding site for this transcription factor at the position –606/–591 (cut-off value: 0.95) suggesting that C/EBPβ may directly regulate S100A4 expression. We also found a putative binding region for the transcription factor AP1 at the –680/–670 position (cut-off value: 0.84) (Figure 2A). It has been described that there are some interactions between C/EBPβ and AP1 in the regulation of gene expression. C/EBPβ can bind to AP1 binding elements as homodimers and activate the transcription of the target gene, whereas its heterodimerization with Fos or Jun leads to an alteration of the DNA binding specificity of C/EBPβ to C/EBPβ DNA binding sites [30].

To provide direct evidence that C/EBPβ is recruited to the endogenous S100A4 promoter during transcription *in vivo*, we performed standard chromatin immunoprecipitation (ChIP) assays, which allow the detection of proteins bound to specific regions of DNA *in vivo*. For these assays we used GL261 cells and a specific antibody against C/EBPβ to precipitate the complex formed by DNA and this transcription factor. ChIP analysis with C/EBPβ antibody showed binding of this transcription factor to the S100A4 promoter at the C/EBPβ and AP1 consensus regions (Figure 2B). As a control of specificity of the assay, binding of C/EBPβ to the housekeeping β-actin locus was not detected. These data indicate that C/EBPβ can interact with two regions of the S100A4 promoter.

We next performed transient transfection experiments to determine whether C/EBPβ regulates the activity of the S100A4 gene by cotransfecting the S100A4 promoter-luciferase construct together with the pcDNA3/C/EBPβ overexpression construct in GL261 cells (Figure 2C). GL261 cells cotransfected with pcDNA3/C/EBPβ plasmid displayed a significant increase in the promoter activity of S100A4. Deletion of both C/EBPβ and AP1 binding sites resulted in a significantly reduced C/EBPβ-induced activity. Subsequently, to assess the functional role of the C/EBPβ and AP1 binding sites in S100A4 promoter regulation, we performed site-specific mutagenesis within these binding regions. We transfected the mutated luciferase reporters into GL261 cells and compared their activity with that of wild-type S100A4 promoter. As shown in Figure 2D, disruption of C/EBPβ or both C/EBPβ and AP1 binding sites significantly attenuated S100A4 promoter activity although this reduction was less evident than that observed with the deletion construct.

### S100A4 overexpression in C/EBPβ-depleted cell line reversed invasion and motility induced by C/EBPβ

**Figure 3:**
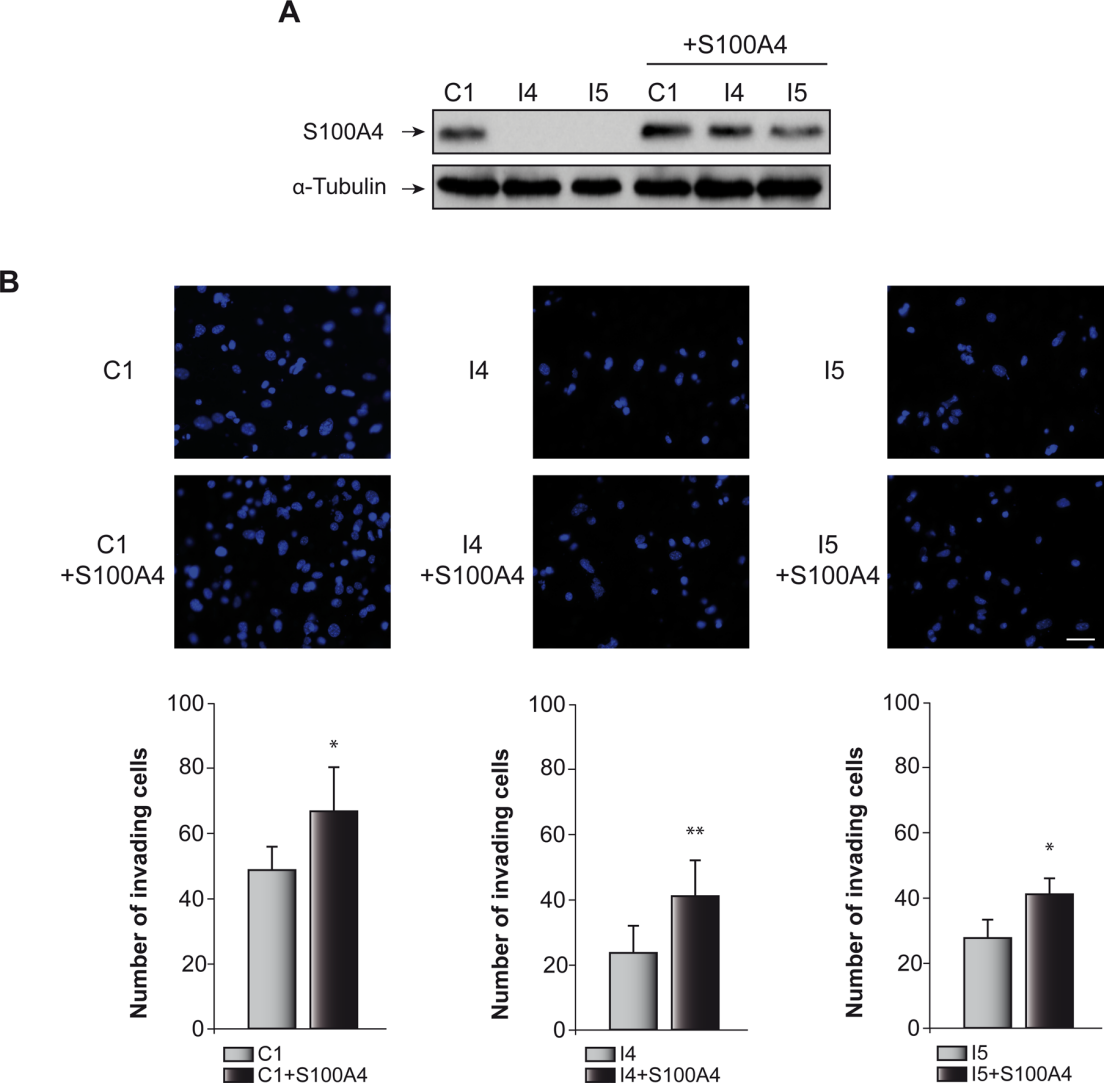
Effect of C/EBPβ and S100A4 on GL261 cells invasion capacity **(A)** Representative Western blots showing S100A4 levels in all the cell lines used for the invasion analysis. **(B)** The invasion capacity of C1, I4 and I5 cells transfected with the S100A4 expressing vector pIRES2-DsRed-Express or the corresponding control vector were determined on transwell chambers coated with Collagen Type IV as described in Methods. Representative images and quantifications are presented. Values are the means ± S.D. of three different experiments. Scale bar 50 μm. **p < 0.01; *p < 0.05.

**Figure 4:**
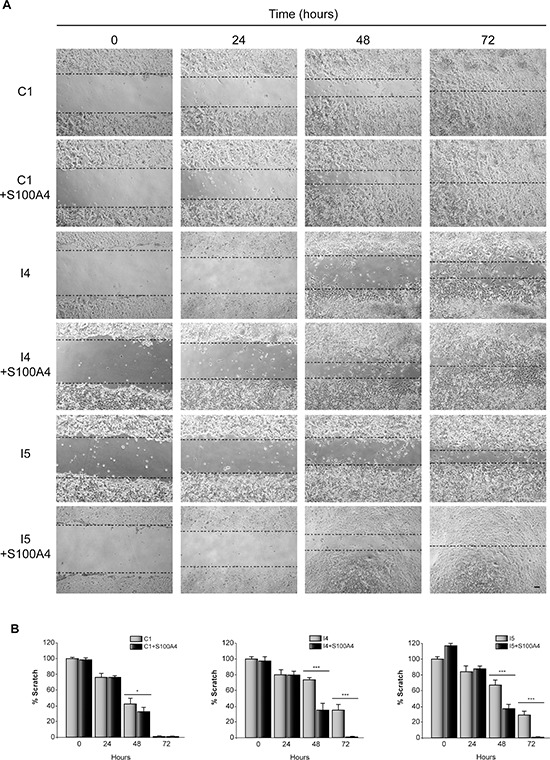
Effect of C/EBPβ and S100A4 on GL261 cells motility C1, I4 and I5 cells transfected with the S100A4 expression vector pIRES2-DsRed-Express or the corresponding control vector were grown until reach confluence. A linear scratch was performed with a plastic pipette tip. Images were taken with a phase contrast microscope at different times after wounding. **(A)** Representative phase-contrast images and **(B)** quantifications of the *in vitro* wound-healing assay are shown. Bar scale 100 μm. ***p < 0.001; *p < 0.05.

Glioblastoma cells are characterized for their capacity to invade normal surrounding tissue. S100A4 is a well-known inductor of tumor cell motility and metastasis in different cancer cells [31], although its role in the invasiveness capacity of glioblastoma cells is not yet known. Previous data from our laboratory, using the “scratch-wound” assay showed that GL261 glioblastoma cells depleted of C/EBPβ presented a restricted cell motility [18]. Here we have analyzed the effect of S100A4 overexpression (Figure 3A) in invasion (transwell assay) and motility (scratch assay) of control and C/EBPβ-depleted GL261glioblastoma cells. As shown in Figure 3B, overexpression of S100A4 caused a clear increased in the invasion capacity of C1, I4 and I5, being more marked in the C/EBPβ-depleted cells. The I4 and I5 cells transfected with the pIRES2-DsRed-Express vector containing S100A4 cDNA showed an increase in their invasion capacity to levels similar to those found in the non-depleted cells (Figure 3B). Regarding cell motility, our results clearly show that the overexpression of S100A4 increased motility in all GL261 cells, C1, I4 and I5 (Figure 4A and 4B). These results are in accordance with the invasion results described above and suggest that S100A4 gene controls the motility of glioblastoma cells and that therefore could mediate the effects of C/EBPβ on motility and invasion.

**Figure 5:**
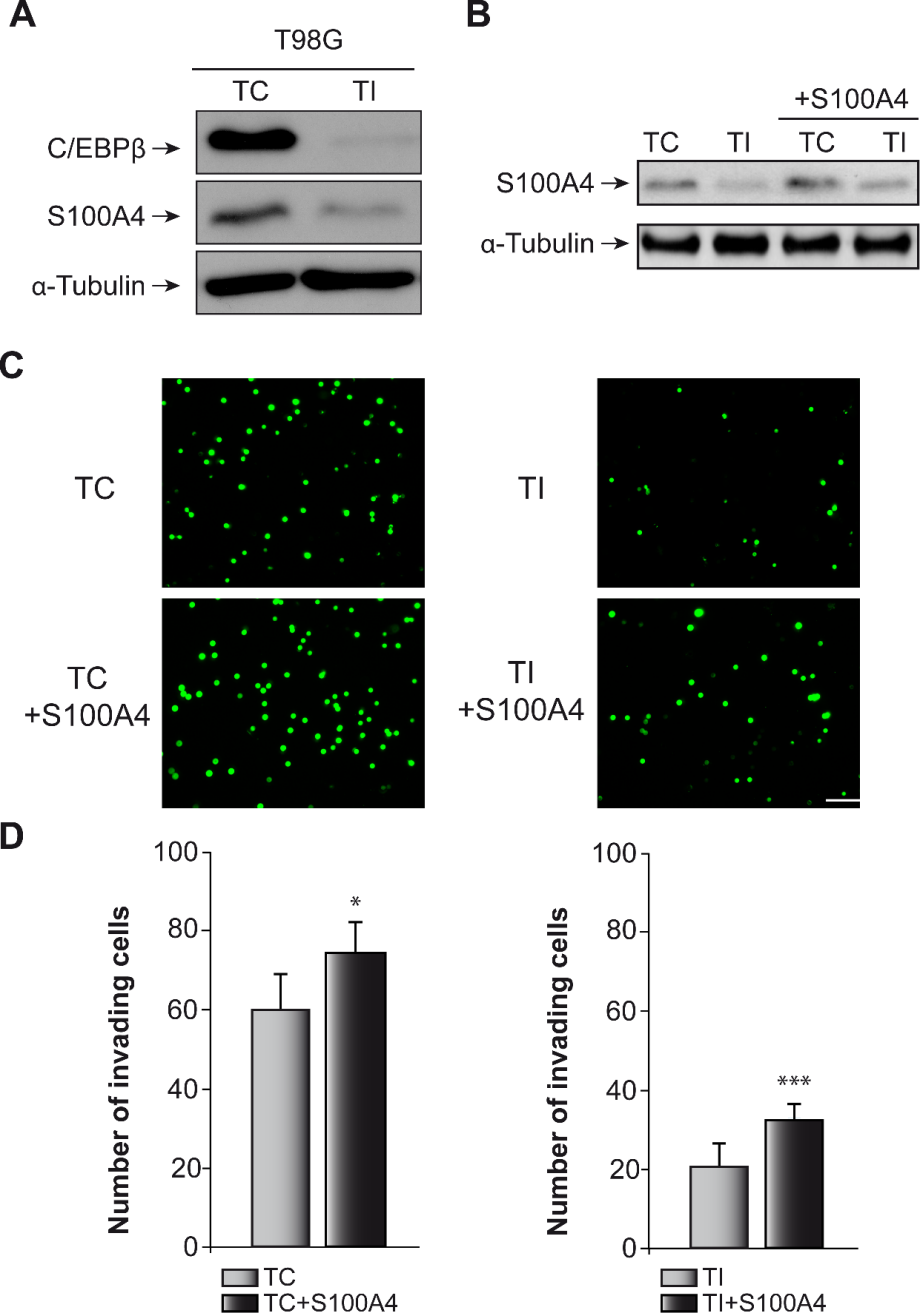
Effect of C/EBPβ and S100A4 on T98G cells invasion capacity **(A)** Representative western blot of C/EBPβ and S100A4 in control (TC) and C/EBPβ interfered (TI) T98G cells. **(B)** Representative western blot showing S100A4 levels in all the cell lines used for the invasion analysis. **(C)** The invasion capacity of TC and TI cells transfected with the S100A4 expressing vector pIRES2-DsRed-Express or the corresponding control vector was determined on transwell chambers coated with Collagen Type IV as described in Materials and Methods. **(C)** Representative images and **(D)** quantifications are presented. Values are the means ± S.D. of three different experiments. Scale bar 50 μm. ***p < 0.001; *p < 0.05.

**Figure 6:**
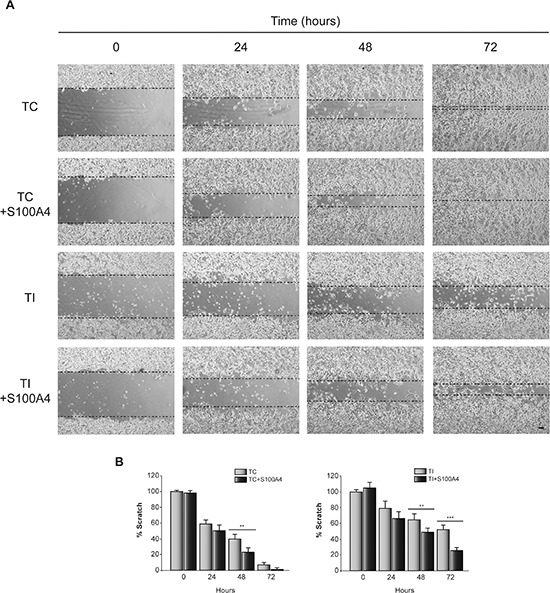
Effect of C/EBPβ and S100A4 on T98G cells motility TC and TI cells transfected with the S100A4 expressing vector pIRES2-DsRed-Express or the corresponding control vector were grown until reach confluence. A linear scratch was performed with a plastic pipette tip. Images were taken with a phase contrast microscope at different times after wounding. **(A)** Representative images and **(B)** quantifications of the *in vitro* wound-healing assay are shown. Bar scale 100 μm. ***p < 0.001; **p < 0.01.

Next, we determined the effect of C/EBPβ depletion and S100A4 overexpression (Figure 5A and 5B) on invasion and motility in the human glioblastoma cell line T98G. Similar to the result observed in GL261 cells, C/EBPβ depletion in T98G cells caused a marked decrease in S100A4 protein content (Figure 5A), invasion capacity (Figure 5C and 5D) and cell motility (Figure 6). The overexpression of S100A4 in T98G cells, as in GL261 cells, increased invasion capacity (Figure 5C) and motility (Figure 6A and 6B) in both non C/EBPβ-depleted (TC) and C/EBPβ-depleted (TI) cells.

Since it has been shown [32, 33] that S100A4 regulates the expression of invasion- and migration-associated genes, such as metalloproteases (MMPs), we next analyzed the mRNA levels of MMP2, which is known to be regulated by S100A4 in esophageal squamous cell carcinoma [34]. Indeed, we found a significant decrease in MMP2 expression in the C/EBPβ-depleted I4 cells (Supplemental Figure S1). These results further confirm the idea that S100A4 is a mediator of the effects of C/EBPβ in the invasion and motility capacities of glioblastoma cells.

**Figure 7:**
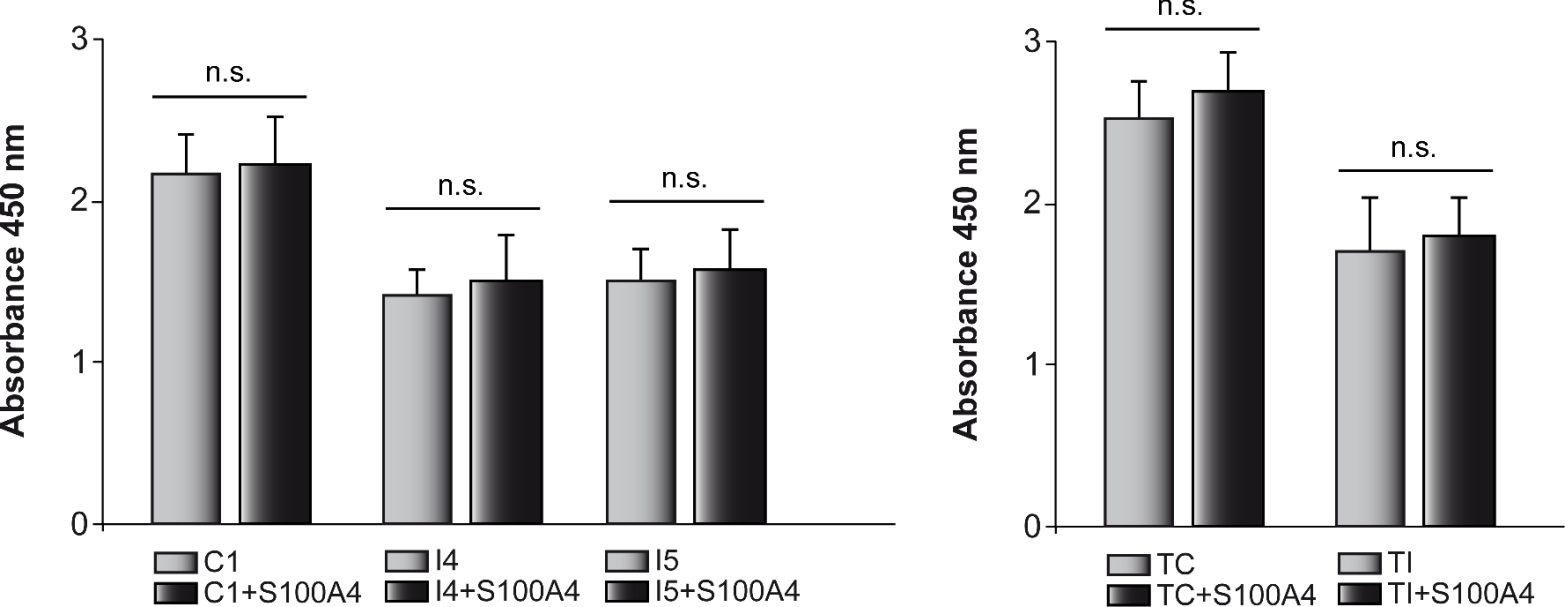
Effects of C/EBPβ and S100A4 expression on cell proliferation The proliferation of mouse (C1, I4 and I5) and human (TC and TI) cells transfected with the S100A4 expressing vector pIRES2-DsRed-Express or the corresponding control vector was determined using *“QIA127 Rapid Cell Proliferation Kit”*, as indicated in Methods. Cells were seeded into individual wells of a 96-well plate and cultivated for 24 h after which WST-1 was added to the culture medium. The cleavage of the WST-1 was quantified by absorbance measurement at 450 nm. Values are the means ± S.D. of three different experiments in triplicate.

In order to know whether S100A4 could also play a role in proliferation of GL261 and T98G cells, we performed *“QIA127 Rapid Cell Proliferation Kit”* assay both in control and C/EBPβ-depleted cells when S100A4 is overexpressed. As can be shown in Figure 7, we didn't find any difference in growth and viability between control and S100A4-overexpressing cells. These results are in agreement with data from Takenaga et al [35] showing that S100A4 is not involved in the growth of C6 glioblastoma cells.

### Effect of C/EBPβ depletion on glioblastoma stem cells

The cancer stem cell (CSC) hypothesis suggests that tumors are organized in a hierarchy with a subpopulation of cells with stem cells properties, responsible for tumor maintenance and progression. Studies from our laboratory indicate that C/EBPβ is implicated in neural stem cells proliferation and differentiation in normal adult mouse brain [8]. In view of these data and based on our previous results showing that C/EBPβ plays an important role in the progression of tumorigenicity of glioblastoma cells, we finally analyzed whether C/EBPβ interference could also exert an antiproliferative effect on glioblastoma stem cells by analyzing its effect in glioblastoma-derived neurospheres (GNSF) and also if S100A4 levels were down-regulated in C/EBPβ-depleted GNSFs.

**Figure 8:**
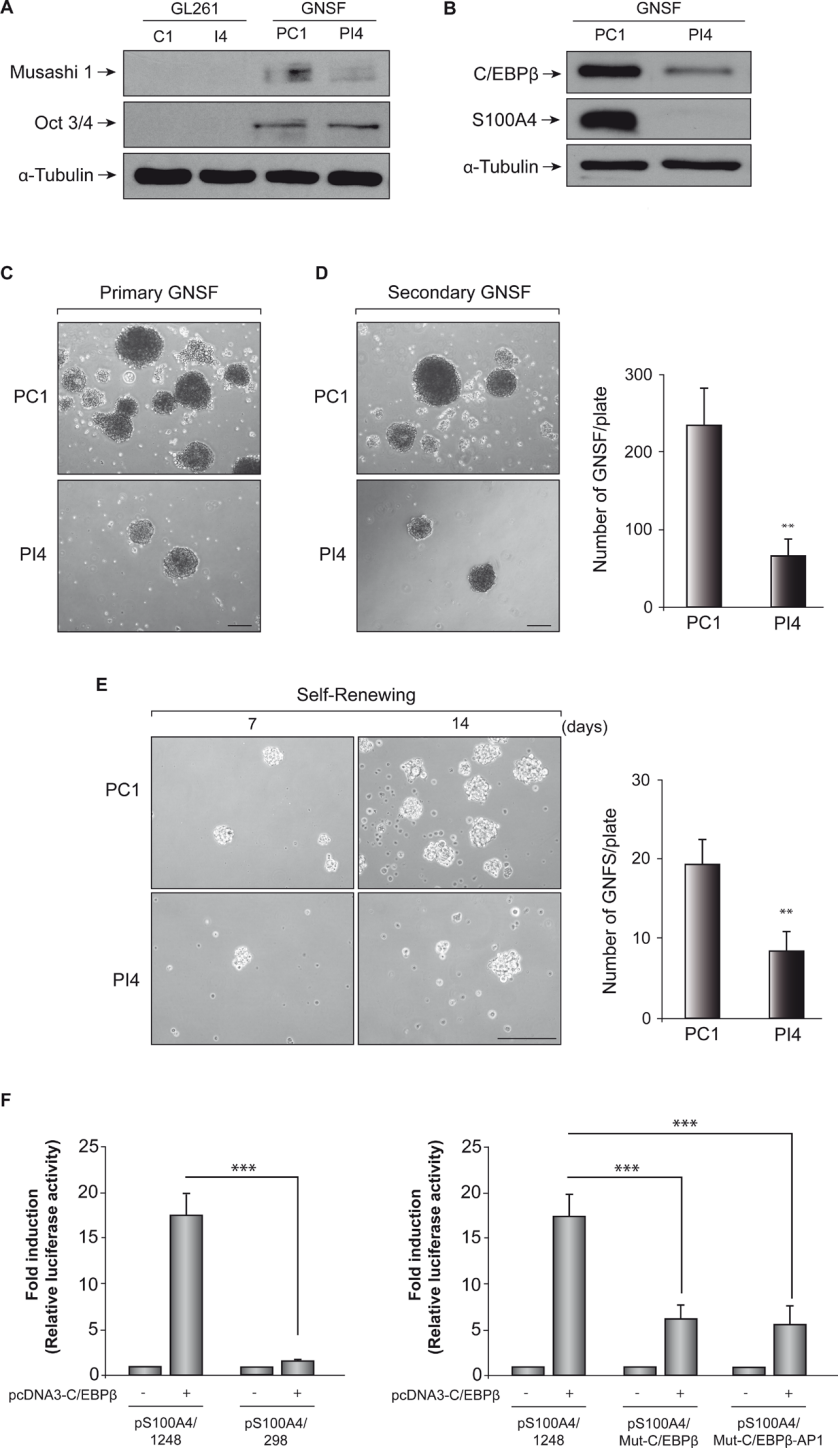
Effect of C/EBPβ depletion on GL261 stem cells **(A)** Representative Western blot showing expression of Musashi-1 and Oct3/4 in adherent C1 and I4 cell lines and neurospheres cultures (GNSF) from PC1 and PI4 7 days after plating. **(B)** Representative Western blot showing expression of C/EBPβ and S100A4 in neurospheres cultures derived from C1 and I4 cells (PC1 and PI4, respectively). **(C)** Representative images of primary GNSF from PC1 and PI4 cells. **(D)** Primary GNSF were dissociated and grown during 14 days to analyze secondary GNSF formation. Quantification of secondary GNSF was performed counting 12 randomly selected fields in 3 different wells. Within each assay the experiments were performed in triplicates. Values represent the means ± SD. **p < 0.01 **(E)** Representative microphotographs showing self-renewal capacity of neurosphere cultures from PC1 and PI4 cells 7 and 14 days after plating. Quantification of new GNSF after 14 days growing was performed counting 12 randomly selected fields in 3 different wells. Scale bar 250 μm. **p < 0.01 **(F)** Transient transfection experiments in GNSF. The entire promoter fragment (pS100A4/1248), a 5′-deletion construct (pS100A4/298), and two constructs containing the mutated C/EBPβ (pS100A4/Mut-C/EBPβ) and the mutated C/EBPβ and AP1 binding sites (pS100A4//Mut-C/EBPβ-AP1) were created as indicated in Methods. Data are expressed relative to the basal values and represent the mean ± SD luciferase activity determined in triplicate in at least three independent experiments. ***p < 0.001.

To ensure that we were working with an enriched population in GNSF, we first analyzed by western blot the protein levels of Musashi-1 and Oct3/4, which are well-known stem cells markers [36]. As expected, adherent C1 and I4 cells did not have Mushashi-1 and Oct3/4 proteins, while GNSF populations of PC1 and PI4 presented detectable levels of these markers (Figure 8A). Also, we observed a decrease in the levels of Musashi-1 in PI4 cells, compared to PC1, suggesting that C/EBPβ can alter the population of stem cells present in these cultures.

Next, we analyzed the basal level of expression of C/EBPβ in murine GNSF populations obtained from control C1 line and C/EBPβ-depleted I4 cells (PC1 and PI4 pools, respectively). Our results show that as happened in adherent C1 and I4 cells, PI4 cultures presented a significant (80%) decrease in the expression levels of C/EBPβ (Figure 8B). This reduction in C/EBPβ levels was accompanied by a large reduction in S100A4 levels, indicating that C/EBPβ is also able to regulate S100A4 expression in the subpopulation of glioblastoma stem cells.

We then studied whether C/EBPβ interference could affect the formation capacity and self-renewal of GNSF population in GL261 glioblastoma cells. Figure 8C shows that C/EBPβ depletion decreases the formation of primary glioblastoma neurospheres. We observed a significant decrease, at 7 days of culture, in the number of PI4 glioblastoma neurospheres, as compared to control PC1 cultures. To analyze more deeply the effect of C/EBPβ depletion on the ability of GBM cells to generate new spheres, actively growing 7 day-old GL261-derived primary GNSF cultures were dissociated, and equal numbers of viable cells were replated in fresh neurosphere medium to generate new GNSF (secondary glioblastoma-derived neurospheres). After 14 days, we observed that the formation of secondary GNSF were significantly less in the C/EBPβ-depleted cells (PI4), compared with control, PC1 (63 ± 21.5 and 235 ± 46.5 per plate, respectively) (Figure 8D). Furthermore, to test for the effect of C/EBPβ interference on the self-renewal of the glioblastoma neurospheres, we dissociated established 7 days-old primary GNSF cultures and plated them at very low density during 7 and 14 days. As shown in Figure 8E, 8C/EBPβ-depleted cultures (PI4) presented less number of secondary GNSF, 7 and 14 days after plating, compared with control population, PC1 (14 days after plating values were 8 ± 2.5 and 19 ± 3.0 per plate, respectively), indicating that these cultures contain less self-renewing stem cells.

Lastly, we evaluated whether the S100A4 promoter was also regulated in the GNSF cultures by C/EBPβ. Consistent with the transfection data using GL261 adherent cells, we found a substantial increase in the basal activity of the pS100A4/1248 construct containing S100A4 promoter in those GNSF co-transfected with the pcDNA3/C/EBPβ overexpression construct (Figure 8F). Also, deletion of C/EBPβ and AP1 binding sites, as well as site-specific mutations in both sites, significantly attenuated C/EBPβ-induced activity of this promoter.

## DISCUSSION

Glioblastomas (GBM) are the most common brain tumor in adults. Currently this devastating disease is practically incurable and the patients show a mean survival time of approximately one year after diagnosis [37], despite the use of surgery, radiotherapy and chemotherapy [38]. Studies of gene expression in glioblastomas have allowed analyzing the transcriptional activity present in these tumors, allowing the classification and prediction of response to particular treatments [39–41].

In a previous work [18] we have demonstrated that C/EBPβ is crucial to regulate glioblastoma cell growth and transformation. These findings are in accordance with the results of Carro et al [42] showing that the expression of C/EBPβ is linked to the mesenchymal state of primary glioblastoma and provides an excellent prognostic biomarker for tumor aggressiveness. These authors showed that C/EBPβ, together with STAT3, act synergistically to initiate and regulate mesenchymal transformation. Overexpression of these transcription factors reprograms neural stem cells throughout the aberrant mesenchymal lineage and enhances tumor aggressiveness.

Also we showed that C/EBPβ regulates several genes involved in DNA repair, invasion and metastasis, suggesting that these genes could be important downstream effectors of C/EBPβ-mediated oncogenic properties [18]. One of these genes is S100A4, which is known to promote invasion and metastasis in different tumor cells [31]. In this regard, Yonemura et al. demonstrated that 55% of gastric cancer patients exhibited elevated S100A4 levels, which were found to be positively associated with high incidence of metastasis. This study also showed that patients with low expression of S100A4 have lower number of metastatic lesions [43]. With respect to glial tumors, it has been shown that S100A4 is expressed differentially in astrocytic tumors being its levels higher on those tumors with a higher degree of malignancy [44].

In the present study, we show that the transcription factor C/EBPβ directly regulates the expression of metastatic protein S100A4 in a mouse glioblastoma cell line and that this regulation may underlie the effects of C/EBPβ in the invasiveness of glioblastoma cells. The expression of S100A4 is very low in both mouse GL261 and human T98G glioblastoma cells depleted of C/EBPβ, which is associated with a decreased in migration and invasiveness capacity of these cells. The migratory and invasiveness activities of these cells was significantly increased in C/EBPβ-depleted cells overexpressing S100A4, suggesting that this protein could be mediating the effects of C/EBPβ on these processes. These results, suggest that S100A4 is a mediator of C/EBPβ effects specifically on cell migration and invasion capacity of glioblastoma cells and are in accordance with previous reports showing a direct relationship between S100A4 expression and cell migration in astrocytic tumor cells, such as C6 [35], neuroblastoma cells [45] and medulloblastoma cells [46]. Our data showing that S100A4 does not enhance proliferation either control or C/EBPβ-depleted cells, suggest that the differences observed were indeed due to differences in cell migration and not to an effect of S100A4 in cell growth.

S100A4 belongs to a large family of calcium binding proteins, many of which are involved in tumorigenic processes [47, 48] including regulation of cell motility, invasion, and migration. In this work we also show that the levels of three of these proteins, S100A6, S100A8, and S100A10, are also regulated by C/EBPβ. This lends support to the view that this transcription factor is an important regulator of different steps of tumorigenic processes in part due to its regulation of different members of the S100 family. Furthermore, these results are in agreement with previous data showing a modulation of the levels of these proteins in different cancer cells [49].

Sequence analysis revealed that the S100A4 promoter contains 2 putative C/EBPβ-binding sequences, which could mediate the transcriptional regulation of this gene by C/EBPβ (one C/EBPβ and one AP1 consensus binding sites). Our data provide evidence that C/EBPβ directly interacts with both sites at the S100A4 promoter regulating its expression. Both, the deletion of a large fragment (almost 1 Kb) of the upstream region containing C/EBPβ and AP1 binding sites, and mutations of the sequences of these sites, clearly reduced C/EBPβ regulation of S100A4 promoter expression. Similar results were obtained when regulation of S100A4 transcriptional activity was evaluated in the GCSC subpopulation. These data, together with the ChIP analysis indicate that C/EBPβ directly regulates S100A4 gene expression. It is interesting to note that it has been reported that after a neural damage, the expression of C/EBPβ [11] and S100A4 [50] is induced, both *in vitro* and *in vivo*.

There is mounting evidence that neural stem cells can be transformed into cancer stem cells and give rise to malignant gliomas by escaping the mechanisms that control proliferation and programmed differentiation [51] [52, 53]. In fact, glioblastomas where among the first solid tumors where a cancer stem cell subpopulation was identified [54]. Here, we have also analyzed whether S100A4 was also present in the GCSC subpopulation and if its expression was also regulated by C/EBPβ, as it happened in adherent glioblastoma cells. Also we studied if C/EBPβ depletion had any effect upon this glioblastoma stem cells subpopulation. Our findings demonstrate that the S100A4 protein is highly expressed in GCSCs and that its expression is completely abolished in those GCSCs depleted of C/EBPβ. Moreover, by using transient tranfection experiments with the S100A4 promoter, we here show that S100A4 is also directly regulated by C/EBPβ in GCSCs. In this regard, it is interesting to note that the abundance of S100A4 has been positively correlated to the self-renewal capability and stemness of different cancer stem cells by other authors [55] [28, 56]. Indeed, Harris et al [28] suggest that S100A4 could be a candidate marker for these cells. The results here obtained confirm the data of these authors and also suggest that C/EBPβ could also be regulating the invasive capacity of CSC through the regulation of the S100A4 gene.

The data presented here show that C/EBPβ interference has a growth inhibitory effect not only upon the bulk of glioblastoma cells, as previously shown by our group [18] but also on the glioblastoma stem cell population. The decreased on C/EBPβ expression results in a reduction of neurospheres formation and expansion in culture, as well as their capacity of self-renewal. These data suggest that C/EBPβ is able to both inhibit the growth of the bulk of the tumor, characterized by actively cycling cells, but also to hinder the growth and motility of glioblastoma stem cells characterized by a low rate of division and high metastatic capacity. This effect of C/EBPβ is probably independent of S100A4 regulation as shown by the lack of effect of S100A4 on cell growth. This effect of C/EBPβ is probably independent of S100A4 regulation as shown by the lack of effect of S100A4 on cell growth.

Overall, our findings suggest that targeting C/EBPβ in glioblastoma cells could have a therapeutic benefit by directly inhibiting the S100A4 gene, which is known to be involved in tumor invasiveness. Our results also point to a role of C/EBPβ on the maintenance of stem cells present in glioblastomas.

## METHODS

### Cell culture and C/EBPβ-shRNA stable transfection

GL261 murine glioblastoma cells were obtained from the NCI-Frederick Cancer Research Tumor Repository (Frederick, MD) and propagated in RPMI medium with 10% fetal bovine serum as described [57]. To knockdown C/EBPβ expression, siRNA sequences against mouse C/EBPβ and a non-targeting siRNA control were obtained from Dharmacon (Thermo Scientific, Waltham, MA). The interfering selected sequence was 5′-GAG CGA CGA GTA CAA GAT GTT CAA GAG ACA TCT TGT ACT CGT CGC TCT T-3′. The oligonucleotides were annealed and the double-stranded oligonucleotides were cloned into pSilencer 4.1vector (Ambion, Austin, TX), in which siRNAs were expressed under the control of the CMV promoter. The construct was verified by DNA sequencing. The plasmids (control or C/EBPβ-shRNA) were transfected into GL261 glioblastoma cells by using *Lipofectamine 2000* (Invitrogen, CA) and pools were selected using 400 μg/ml of G418 and maintained in this selection medium. Pools C1 (expressing a non-targeting siRNA control) and I4 (expressing a siRNA against C/EBPβ) [18] were used throughout the study. T98G human glioblastoma cells were obtained from the ATCC (American Type Culture Collection) and propagated in EMEM medium with 10% fetal bovine serum. C/EBPβ expression was silenced in T98G cells using the FUGW lentiviral vector (obtained from Dr. Quintanilla-Martinez, Department of Dermatology, Eberhard Karls University, Tübingen-Germany). The interfering selected sequence was: 5′-GAAGACCGTGGACAAGCAC-3′ (pool TI). A non-targeting sequence (5′-GCCGCTTTGTAGGATAGAG-3′, pool TC) was used as control. In addition to the interfering and control sequences, the lentiviral vectors also express the GFP protein. For lentiviral production, 293T cells were transiently transfected with the appropriate lentiviral expression vector and the vectors pMD2-G, pMDLg/pRRE, and pRSV-Rev, which encode lentiviral proteins. The medium containing the lentiviruses was recovered, filtered through a 0.45-μm filter and used to infect T98G cells. The infection was repeated 8 h and 24 h later. Pools TC (expressing a non targeting shRNA) and TI (expressing a shRNA against C/EBPβ) were used in this study.

### Quantitative real-time PCR

Total RNA was extracted from C1, I4 and I5 pools by using TRIzol (Invitrogen, Carlsbad, CA) as recommended by the manufacturer. Complementary DNA was generated using Superscript III kit (Invitrogen). PCR was performed on ABI Prism 7700 Sequence Detector (Applied Biosystems) using TaqMan probes (Applied Biosystems) specific to S100A4 and β-actin, following the manufacturer's protocol. S100A6, S100A8, and S100A10 and MMP2 mRNA levels were analyzed by qRT-PCR using Fast SYBR Green Master Mix (Applied Biosystem) and 300 nM concentrations of specific primers (listed in Supplemental Table SI). In all samples, each specific sequence was measured at least twice in triplicate. Data were analyzed using the 2^−ΔΔCt^ method [58] and the housekeeping β-actin for normalization.

### Immunoblot analysis

Cultured cells were harvested and lysed in ice-cold RIPA buffer and equal quantities of total protein were separated by 10% SDS-PAGE. After electrophoresis, proteins were transferred to nitrocellulose membranes (Protran, Whatman, Dassel, Germany) and blots were probed with the indicated primary antibodies, as previously described (Cortes-Canteli et al., 2004). The antibodies used were the following: rabbit polyclonal anti-C/EBPβ, rabbit polyclonal anti-Oct3/4 (Santa Cruz Biotechnology, CA), rabbit polyclonal anti-S100A4 (Abcam), rabbit polyclonal anti-Musashi-1 (Abcam) and mouse monoclonal anti-α-tubulin (Sigma). Secondary peroxidase-conjugated donkey anti-rabbit and rabbit anti-mouse antibodies were from Amersham Biosciences (GE Healthcare, Buckinghamshire, England) and Jackson Immunoresearch, respectively. The Western blots shown in Figures 1, 3, 5 and 8 are representative of at least three independent experiments.

### Chromatin immunoprecipitation

ChIP analysis was performed essentially as described [59]. For immunoprecipitation, the following antibodies were used: 5 μg of rabbit polyclonal anti-C/EBPβ and 2 μg of normal rabbit IgG (Santa Cruz Biotechnology, CA). The precipitated DNA was analyzed by PCR using the following primers:

C/EBPβ, sense: 5′-TCCTGACTCCCCCTTTTAC C-3′; antisense: 5′-GGAGGCCAT GATGGAGTTAG-3′. AP-1, sense: 5′-CCCCGAATTTGTACCCTATC-3′; antisense: 5′-AGCATTCGGGGTTGAATGT-3′. β-actin, sense: 5′-TTGTAACCAACTGGG ACGACATGG-3′; antisense 5′-GATCTTGATCTTCATGGTGCTAGG-3′.

### Generation of mouse S100A4 promoter constructs

Mouse S100A4 promoter was PCR-amplified from mouse genomic DNA using the high fidelity PCR-Extender System DNA polymerase (5-Prime, Gaithersburg, MD). PCR reactions were performed according to the manufacturer's recommendations. The amplified products, a long promoter fragment from positions –1248 to +267, (pS100A4/1248) and a 5′-deletion construct of the pS100A4/1248 promoter, from positions –298 to +267, (pS100A4/298) were cloned in the promoterless luciferase reporter vector pGL4.10. The primers used are listed in Supplementary Table SII. Mutant constructs of mouse S100A4 promoter were generated with the Quick Change Site-Directed Mutagenesis Kit (Stratagene, La Jolla, CA), using the S100A4 promoter cloned in the reporter plasmid pGL4.10 as a template. Specific mutations were incorporated by polymerase chain reaction using *Pfu* Turbo DNA Polymerase and subsequent digestion with *DpnI*, to eliminate the methylated parental band. The sequences were confirmed by sequencing. The mutants generated were pS100A4/Mut-C/EBPβ, where the C/EBPβ binding site at –606/–591 was mutated (from TGTCATTCCTCAATATC to TGAGCGGGGGGCCGCAC) and pS100A4/Mut-C/EBPβ-AP1 where both, the C/EBPβ (as previously indicated) and the AP1 binding sites at 680/−670 (from CCTGACTCCCC to GGGCCGGGGGG) are mutated.

### Transient transfections

For transient transfection experiments, semi-confluent GL261 cells were transfected with *lipofectamine 2000* (Invitrogen) using different constructs: pS100A4/1248 (complete promoter), pS100A4/Mut-C/EBPβ, pS100A4/Mut-C/EBPβ-AP1 or pS100A4/298 (a deletion construct lacking the consensus C/EBPβ- and AP1-binding sites: −298/+267) in the presence or absence of pcDNA3-C/EBPβ (C/EBPβ overexpression plasmid). Forty-eight hours after transfection, cells were harvested for determination of luciferase activity by using a reporter assay system (Promega, Madison, WI). β-galactosidase was used to determine transfection efficiency. Each transient transfection experiment was repeated at least three times in triplicate.

### Cell invasion, migration and wound healing assay

The plasmid pIRES2-DsRed-Express containing S100A4 cDNA obtained from Dr. Stephan Lorenz (Faculty of Medicine, University of Leipzig, Germany) was transfected into mouse GL261 (C1, I4 and I5) and human T98G (TC and TI) cells to overexpress S100A4 protein.

Tumor cell invasion assays were performed using Transwells chambers (Costar) with 12-μm (mouse cells) or 8-μm pores (human cells) coated with a layer of Collagen type IV (50 μg/ml) free of growth factors (BD Biosciences). Medium with 20% fetal bovine serum was added to the lower chambers of the Transwells. Mouse (C1, I4 and I5) and human cells (TC and TI) were seeded (50,000 and 150,000 cells respectively) on top of the Transwell in triplicate in medium without serum and incubated at 37º for 48 h. At the end of the experiments, the bottom filters were fixed and stained with DAPI in the case of mouse cells. For human cells, GFP was used for cell quantification. Cells in the top chambers were removed by wiping with cotton swabs, and the stained cells that had migrated through the Collagen IV were counted under a microscope. Ten randomly selected 20x microscopic fields were counted using the ImageJ program.

Wound healing assay was used to detect the alteration of cell motility. The different cells lines were seeded onto 60-mm plates and, after overnight incubation, an artificial wound was created using a P200 pipette tip to scratch on the confluent cell monolayer. Photomicrograph was taken immediately (time 0 h), so that the migrating cells and closing of scratch wound could be observed. Microphotographs were also taken at 24, 48 and 72 h post-wounding. Within each assay the experiments were performed in triplicate.

Transfection efficiency using lipofectamine 2000 (as measured by the percentage of red-fluorescent cells) was ~60%.

### Cell proliferation assay

The effects of C/EBPβ and S100A4 expression on cell proliferation were determined using the *“QIA127 Rapid Cell Proliferation Kit”* (Calbiochem), according to the manufacturer's protocol. Mouse and human cells transfected or not with the plasmid pIRES2-DsRed-Express overexpressing S100A4 were seeded in triplicate onto 96-well plates at a density of 10,000 cells/well for mouse cells and 20,000 cells/well for human cells. After 24 h of growth, cells were treated with 10 μl of the tetrazolium salt WST-1 labeling mixture, to each well. Cells were incubated for 2 h at 37ºC. The cleavage of the WST-1 was quantified by absorbance measurement at 450 nm.

### Glioblastoma neurosphere (GNSF) formation

For GNSF formation C1 and I4 cells were plated and grown in regular medium (RPMI, 10% FBS, glutamine, gentamicine and fungizone). Two days after plating, supernatant was collected, centrifuged and cells were replated in a defined serum-free tumor sphere Ham's F-12/Dulbecco's modified Eagle's medium (1:1) supplemented with B27 (Invitrogen, Carlsbad, CA), 20 ng/ml epidermal growth factor (EGF, Peprotech, EC) and 20 ng/ml fibroblast growth factor (FGF, Peprotech, EC). After 1 week in culture some primary GNSF control (PC1) and C/EBPβ-depleted (PI4) were formed. These primary GNSF were then dissociated, and 50,000 cells/ml were replated in proliferative conditions and grown for another 14 days to score the number of secondary neurospheres generated. For self-renewing experiments, primary glioblastoma neurospheres were dissociated and plated at a density of 2,000 cells/ml for another 7 or 14 days in proliferative medium. These assays were repeated at least three times in triplicate.

For reverse transfection experiments, cultured GNSF were used. A reverse transfection protocol was performed to deliver pS100A4/1248 (complete promoter), pS100A4/Mut-C/EBPβ, pS100A4/Mut-C/EBPβ-AP1 or pS100A4/298 (a deletion construct lacking the consensus C/EBPβ- and AP1-binding sites: −298/+267) in the presence or absence of pcDNA3-C/EBPβ (C/EBPβ overexpression plasmid) into GNSFs. Briefly, a transfection complex was prepared by diluting constructs (final plasmid concentration: 1 μg/well) in 100 μL OPTI-MEM (Invitrogen), then adding 100 μL OPTI-MEM containing 1 μL Lipofectamine 2000 transfection reagent (Invitrogen). Neurospheres were disaggregated and 2 × 10^4^ cells seeded in a 24-well plate, and transfected with the above-mentioned complex. Forty-eight hours after transfection, cells were harvested for determination of luciferase activity by using a reporter assay system (Promega, Madison, WI). β-galactosidase was used to determine transfection efficiency. Each transfection experiment was repeated at least three times in triplicate.

## SUPPLEMENTARY FIGURE AND TABLES


