# Supplementary material for: CCAAT/Enhancer binding protein β induces motility and invasion of glioblastoma cells through transcriptional regulation of the calcium binding protein S100A4
Source: Oncotarget. 2015 Jan 24;6(6):4369–84. doi: 10.18632/oncotarget.2976 (PMC4414196; doi:10.18632/oncotarget.2976)
Supplement: Supplementary file 1 [file oncotarget-06-4369-s001.pdf]

SUPPLEMENTARY FIGURE AND TABLES

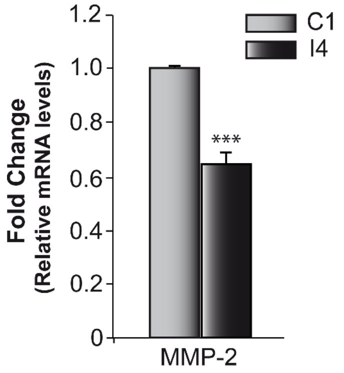

Supplemental Figure S1: Quantification of MMP-2 mRNA levels in GL261 control cell line (C1) and C/EBPβ-depleted (I4) cells by quantitative real time-PCR. As indicated in Methods, we used Fast SYBR Green and primers specifics to MMP-2 and mouse β-Actin. The graphic shows the means of values $2^{-\Delta\Delta Ct}$ MMP-2/β-actin ± SD. ****p* < 0.001.

Supplemental Table SI: Primers used for real-time quantitative real time-PCR

Name	Forward Primers Sequence (5′ to 3′)	Reverse Primers Sequence (5′ to 3′)
S100A6	CGCTTCTTCTAGCCCAGTCAT	ACTGGATTTCACCGAGAGAGG
S100A8	GGAAATCACCATGCCCTCTACAA	ATGCCACACCCACTTTTATCACC
S100A10	AGACCACTTCACAAAGGAGGAC	GCCTATTTCTTCCCCTTCTGCT
MMP-2	CTTCGCTCGTTTCCTTCAAC	AGAGTGAGGAGGGGAACCAT
β-Actin	GACGGCCAGGTCATCACTAT	ACATCTGCTGGAAGGTGGAC

Supplemental Table SII: Primers used to amplify mouse S100A4 promoter constructs

Constructs	Forward Primers Sequence (5′ to 3′)	Reverse Primers Sequence (5′ to 3′)	Fragment promoter positions
pS100A4/1248	GCTACCCATGAGCTTTCGAG	CTACATCTGAGCTCCCGGAG	–1248 to +267
pS100A4/298	CACACCTCATGTCCAGGTTG	CTACATCTGAGCTCCCGGAG	–1248 to +267
